# re3data – Indexing the Global Research Data Repository Landscape Since 2012

**DOI:** 10.1038/s41597-023-02462-y

**Published:** 2023-08-29

**Authors:** Heinz Pampel, Nina Leonie Weisweiler, Dorothea Strecker, Michael Witt, Paul Vierkant, Kirsten Elger, Roland Bertelmann, Matthew Buys, Lea Maria Ferguson, Maxi Kindling, Rachael Kotarski, Vivien Petras

**Affiliations:** 1 Humboldt-Universität zu Berlin, Berlin School of Library and Information Science, Berlin, Germany; 2 Helmholtz Association, Helmholtz Open Science Office, Potsdam, Germany; 3 University of Purdue, Distributed Data Curation Center, West Lafayette, IN USA; 4 DataCite - International Data Citation Initiative e. V, Hannover, Germany; 5 GFZ German Research Centre for Geosciences, Library and Information Services, Potsdam, Germany; 6 Freie Universität Berlin, Open-Access-Büro Berlin, Berlin, Germany; 7 University of Bath, Library, Bath, UK

**Keywords:** Databases, Publishing

## Abstract

For more than ten years, re3data, a global registry of research data repositories (RDRs), has been helping scientists, funding agencies, libraries, and data centers with finding, identifying, and referencing RDRs. As the world’s largest directory of RDRs, re3data currently describes over 3,000 RDRs on the basis of a comprehensive metadata schema. The service allows searching for RDRs of any type and from all disciplines, and users can filter results based on a wide range of characteristics. The re3data RDR descriptions are available as Open Data accessible through an API and are utilized by numerous Open Science services. re3data is engaged in various initiatives and projects concerning data management and is mentioned in the policies of many scientific institutions, funding organizations, and publishers. This article reflects on the ten-year experience of running re3data and discusses ten key issues related to the management of an Open Science service that caters to RDRs worldwide.

## Introduction

In the 2010s, making research data publicly accessible gained importance: Terms such as e-science^1^ and cyberscience^2^ were shaping discourses about scientific work in the digital age. Various discussions within the scientific community^3–8^ resulted in an increased awareness of the value of permanent access to research data. Policy recommendations of the Organization for Economic Co-operation and Development (OECD)^9^ or the European Commission^10^ reflected this shift.

The need for professional data management was increasingly emphasized with the publication of the now widely recognized *FAIR Data Principles*^11^. Researchers, academic institutions, and funders started to address this issue in policies^12^, initiatives and networks^13–15^, and infrastructures^16–19^. For example, the National Science Foundation (NSF) in the United States published a Data Sharing Policy in 2011, in which the funding agency required beneficiaries to provide information about data handling in a Data Management Plan^20^. In Germany, the German Research Foundation (DFG) published a similar statement regarding access to research data in the 2010s^21,22^.

The handling of research data was also discussed in library and computing center communities: In 2009, the German Initiative for Networked Information (DINI), a network of information infrastructure providers, published a position paper on the need for research data management (RDM) at higher education institutions^23^. Through the discussions within DINI, the need for a registry of RDRs became evident. At the time, the Directory of Open Access Repositories (OpenDOAR)^24^ had already established itself as a directory of subject and institutional Open Access repositories. However, there was no comparable directory for RDRs, and it remained unclear how many repositories dedicated to research data existed.

In 2011, a consortium of research institutions in Germany submitted a proposal to the German Research Foundation (DFG), asking for funding to develop ‘re3data – Registry of Research Data Repositories’^25^. Members of the consortium were the Karlsruhe Institute of Technology (KIT), the Humboldt-Universität zu Berlin, and the Helmholtz Open Science Office at the GFZ German Research Centre for Geosciences. The DFG approved the proposal in the same year. The project aimed to develop a service that would help researchers identify suitable RDRs to store their research data. re3data went online in 2012, and already listed 400 RDRs one year later^26^.

While working on the registry, the project team in Germany became aware of a similar initiative in the USA. With support from the Institute of Museum and Library Services, Purdue and Pennsylvania State University libraries developed Databib, a ‘curated, global, online catalog of research data repositories’^27^. Databib went online in the same year^28^. At the time, RDRs were indexed and curated by library staff at re3data partner institutions, whereas Databib had established an international editorial board to curate RDR descriptions^27^. Databib and re3data signed a Memorandum of Understanding in 2012, and, following excellent cooperation, the two services merged in 2014^29^. The merger brought together successful ideas from each service: The metadata schemas were combined, resulting in version 2.2 of the re3data metadata schema^30^, and the sets of RDR descriptions were merged. The international editorial board of Databib was expanded to include re3data editors. Development of the IT infrastructure of re3data continued, combining the expertise both services had built. For operating the service, a management duo was installed, comprising a member each from institutions representing re3data and Databib.

The two services have always been closely corresponding with DataCite, an international not-for-profit organization that aims to ensure that research outputs and resources are openly available and connected so that their reuse can advance knowledge across and between disciplines, now and in the future^31^. In this process, the main objective was to cover the interests of the global community of operators more comprehensively. In 2015, the DataCite Executive Board and the General Assembly decided to enter into an agreement with re3data, making re3data a DataCite partner service^29^. In 2017, re3data won the Oberly Award for Bibliography in the Agricultural or Natural Sciences from the American Libraries Association^32^.

Today, re3data is the largest directory of RDRs worldwide, indexing over 3,000 RDRs as of March 2023. re3data is widely used by academic institutions, funding organizations, publishers, journals, and various other stakeholders, such as the European Open Science Cloud (EOSC) and the National Research Data Infrastructure in Germany (NFDI). re3data metadata is also used to monitor and study the landscape of RDRs, and it is reused by numerous tools and services. Third-party-funded projects support the continuous development of the service. Currently, the DFG is funding the development of the service within the project re3data COREF^33,34^. In addition, the project partners DataCite and KIT bring the re3data perspective into EOSC projects such as FAIRsFAIR (completed)^35^ and FAIR-IMPACT^29^.

This article outlines the decade-long experience of managing a widely used registry that supports a diverse and global community of stakeholders. The article is clustered around ten key issues that have emerged over time. For each of the ten issues, we first present a brief definition from the perspective of re3data. We then describe our approach to addressing the issue, and finally, we offer a reflection on our work.

## Results

The section outlines ten key issues that have emerged in the last ten years of operating re3data.

### Openness

#### Scope

For re3data, Open Science means providing unrestricted access to the re3data metadata and schema, transparency of the indexing process, as well as open communication with the community of global RDRs.

#### Approach

At all times, re3data has been committed to Open Science by striving to be transparent and by sharing metadata. The openness of re3data pertains not only to the handling of its metadata and the associated infrastructure, but also to collaborative engagements with the community of research data stewards and other stakeholders in the field of research data management.

An example of this is the development of the re3data metadata schema: The initial version of the schema integrated a request for comments that allowed stakeholders to offer suggestions and improvements^26^. This participatory approach, accompanied by a public relations campaign, has yielded positive outcomes. Numerous experts engaged in the request for comments and contributed their perspective and expertise. Based on the positive feedback, we subsequently integrated a participatory phase in further updates of the metadata schema^30,36^.

In addition to this general commitment to openness, re3data has made its metadata available under the Creative Commons deed CC0. Due to adopting this highly permissive license, re3data metadata is strongly utilized by other parties, thereby enabling the development of new and innovative services and tools. Moreover, adaptable Jupyter Notebooks^37^ have been published to facilitate the use of the re3data metadata. Additionally, workshops^38^ have been arranged to support individuals in working with the notebooks and re3data data in general.

As a registry of RDRs, re3data also promotes Open Science by helping researchers find suitable repositories for publishing their data. For researchers who are looking for a repository that supports Open Science practices, re3data offers concise information on repository openness via its icon system. A recent analysis showed that most repositories indexed in re3data are considered ‘open’^39^.

#### Lessons learned

The extensive reuse of re3data metadata increases its overall value, and participatory phases allow for incorporating different perspectives and experiences.

### Quality assurance

#### Scope

For re3data, quality assurance encompasses all processes to ensure a service that meets the needs of a global community, as well as verifiably high-quality information.

#### Approach

**Fig. 1:**
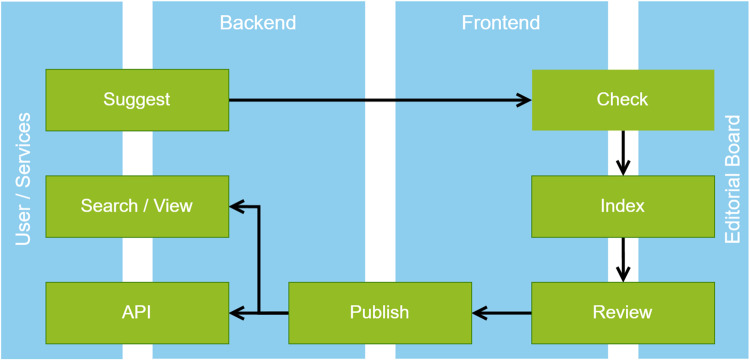
Schematic overview of the editorial process in re3data.

High-quality RDR descriptions are at the core of re3data. Therefore, continuous efforts ensure that re3data metadata describes appropriately and correctly. Figure 1 shows the editorial process in re3data. Anyone, for example RDR operators, can submit repositories to be indexed in re3data by providing the repository name, URL, and some other core properties via a web form^40^. The re3data editorial board analyzes if the suggested RDR conforms with the re3data registration policy^40^. The policy requires that the RDR is operated by a legal entity, such as a library or university, and that the terms of use are clearly communicated. Additionally, the RDR must have a focus on storing and providing access to research data. If an RDR meets these requirements, it is indexed based on the re3data metadata schema. A member of the editorial board creates an initial RDR description, which is then reviewed by another editor. This approach has proven effective in resolving any inconsistencies in interpreting RDR characteristics. An indexing manual explains how the schema is to be applied and helps to ensure consistency between RDR descriptions. Once this review is complete, the RDR description is made publicly visible.

**Fig. 2:**
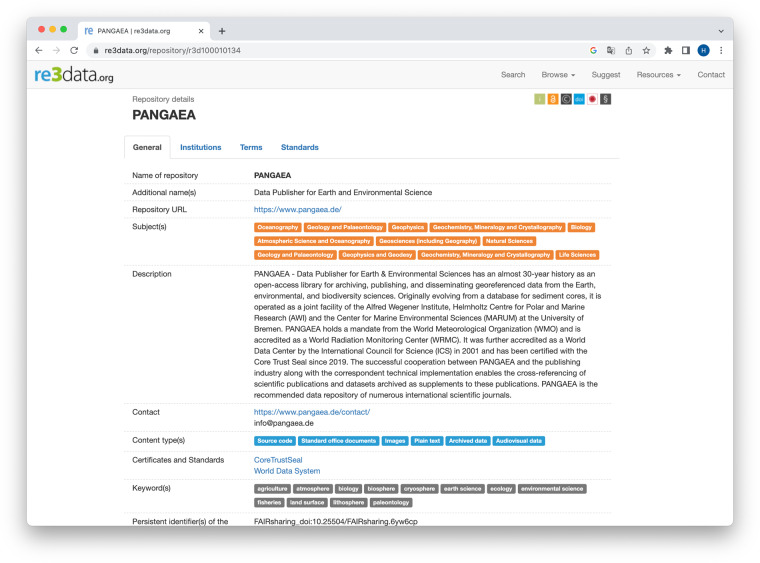
Screenshot of the re3data description of the research data repository PANGAEA^97^.

re3data applies a number of measures to ensure the long-term quality and consistency of RDR descriptions, including automated quality checks. For example, it is periodically checked whether the URLs of the RDR still resolve – if not, the entry of a RDR is reexamined. Figure 2 shows a screenshot of a re3data RDR description.

The re3data metadata schema on which RDR descriptions are based is reviewed and updated regularly to ensure that users’ changing information needs are met. Operators of an RDR, as well as any other person, can suggest changes to RDR descriptions by submitting a change request. A link for filing a change request can be found at the bottom of each RDR description in re3data. Once a change request has been submitted, a member of the editorial board will review the proposed changes and verify them against information on the RDR website. If the change request is deemed valid, the RDR description will be adapted accordingly.

As part of the project re3data COREF, quality assurance practices at RDRs were systematically investigated. The aim was to understand how RDRs ensure high-quality data, and to better reflect these measures in the metadata schema. The results of the study^41^, which were based on a survey among RDR operators, show that approaches to quality assurance are diverse and depend on the mission and scope of the RDR. However, RDRs are key actors in enabling quality assurance. Furthermore, there is a path dependence of data review on the review process of textual publications. In addition to the study, a workshop^42,43^ was held with CoreTrustSeal that focused on quality assurance measures RDRs have implemented. CoreTrustSeal is a RDR certification organization launched in 2017 that defines requirements for base-level certification for RDRs^44^.

#### Lessons learned

Combining manual and automated verification was shown to be most effective in ensuring that RDR descriptions remain consistent while meeting users’ diverse information needs.

### Community engagement

#### Scope

For re3data, community engagement encompasses all activities that ensure interaction with the global RDR community in a participatory process.

#### Approach

Collaboration has always been a central principle for re3data. This is reflected in the fact that research communities, RDR providers, and other relevant stakeholders contribute significantly to the completeness and accuracy of the re3data metadata as well as its further technical and conceptual development. Examples include the participatory phase during the revision of the metadata schema, the involvement of important stakeholders in the development of the *re3data Conceptual Model for User Stories*^45,46^, or the activities that investigate data quality assurance at RDRs.

re3data engages in collaborations in various forms with diverse stakeholders, for example:

- In collaboration with the Canadian Data Repositories Inventory Project and later with the Digital Research Alliance of Canada, both initiatives aiming at describing the Canadian landscape of RDRs comprehensively, descriptions of Canadian RDRs in re3data were improved, and additional RDRs were indexed^47,48^.
- A collaboration initiative was initiated in Germany with the Helmholtz Metadata Collaboration (HMC). In this initiative, the descriptions of research data infrastructures within the Helmholtz Association are being reviewed and enhanced^49^.
- re3data also engages in international networks, particularly within the Research Data Alliance (RDA). Activities focus on several RDA working and interest groups^50–52^ that touch on topics relevant to RDR registries.

#### Lessons learned

Combining strategies of engagement connects the service to its stakeholders and creates opportunities for collaboration and innovation.

### Interoperability

#### Scope

For re3data, interoperability means facilitating interactions and metadata exchange with the global RDR community by relying on established standards.

#### Approach

Interoperability is a necessary condition to integrate a service into a global network of diverse stakeholders. International standards must be implemented to achieve this, for example with the re3data API^53^. The API can be used to query various parameters of an RDR as expressed in the metadata schema. The API enables the machine readability and integration of re3data metadata into other services. The re3data API is based on the RESTful API concept and is well-documented. Applying the HATEOAS principles^54^ enables the decoupling of clients and servers, and thus allows for independent development of server functionality. This results in a robust interface that promotes interoperability and reduces barriers to future use. Also, re3data supports OpenSearch, a standard that enables interaction with search results in a format suitable for syndication and aggregation.

Interoperability also guides the development of the metadata schema: Established vocabularies and standards are used to describe RDRs wherever possible. Examples of standards used in the metadata schema include:

- ISO 639-3 for language information, for example a RDR name
- ISO 8601 for the use of date information on a RDR
- DFG Classification of Subject Areas for subject information on a RDR

In addition, re3data pursues interoperability by jointly working on a mapping between the DFG Classification of Subject Areas used by re3data and the OECD Fields of Science classification used by DataCite^55^.

**Fig. 3:**
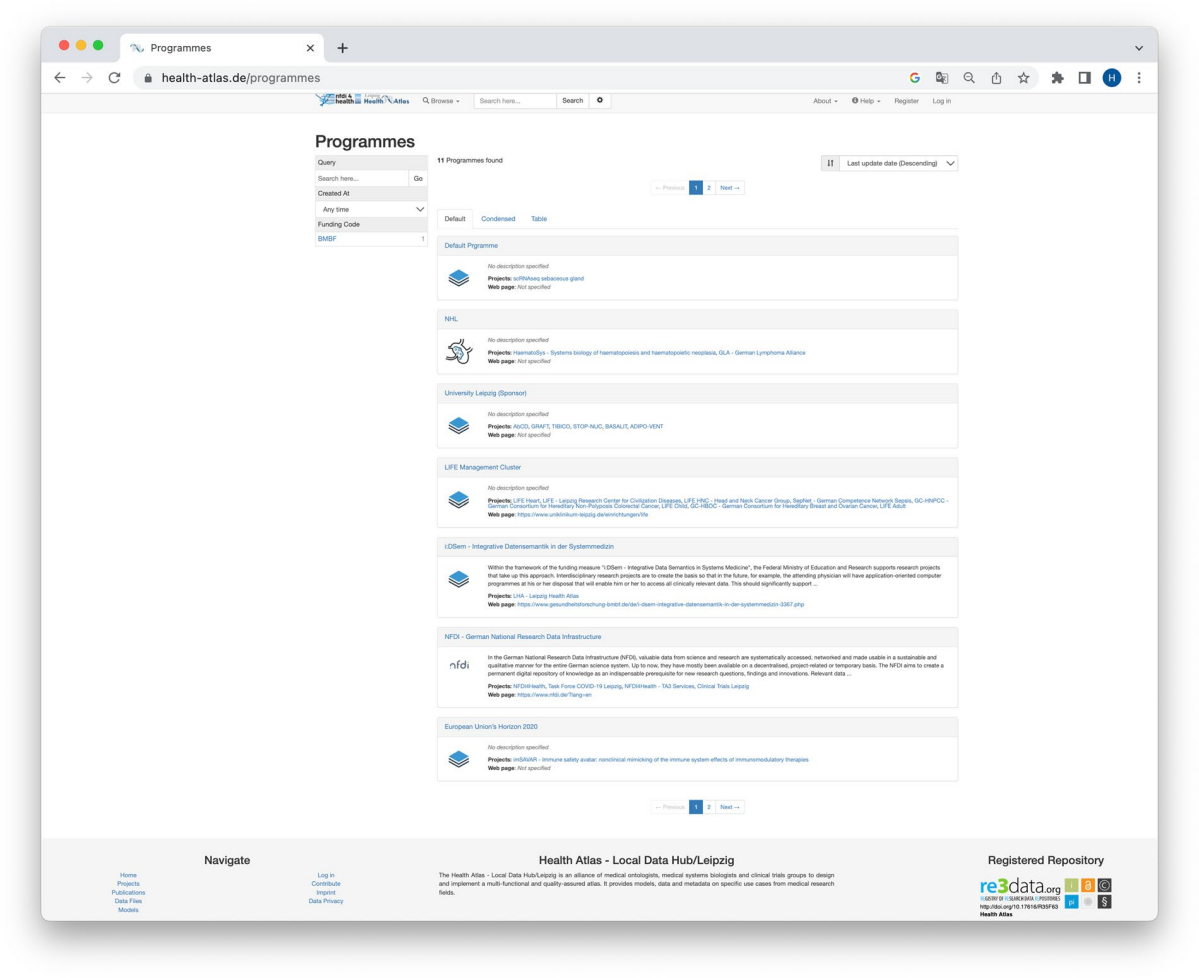
The re3data badge integrated in the research data repository Health Atlas.

re3data records whether an RDR has obtained formal certification, for example by World Data System (WDS) or CoreTrustSeal. The certification status, along with other properties, is visualized by the re3data icon system that makes the core properties of RDRs easily accessible visually. The icon system provides information about the openness of the RDR and its data collection, the use of PID systems, as well as the certification status. The icon system can also be integrated into RDR websites via badges^56^. Figure 3 shows an example of a re3data badge.

re3data captures information that might be relevant to metadata aggregator services, including API URLs, as well as the metadata standard(s) used. In offering this information in a standardized form, re3data fosters the development of services that span multiple collections, such as data portals. For example, as part of the FAIRsFAIR project work, re3data metadata has been integrated into DataCite Commons^57^ to embed repository information in the DataCite PID Graph. This step not only improves the discoverability of repositories that support research data management in accordance with the FAIR principles but also serves as a basis for the development of new services such as the FAIR assessment tool F-UJI^35,58^.

#### Lessons learned

The adherence to established standards facilitates the reuse of re3data metadata and increases the integration of the service into the broader Open Science landscape.

### Developement

#### Scope

For re3data, continuous development ensures that the service is able to respond dynamically to evolving requirements of the global RDR community.

#### Approach

Maintaining a registry for an international community poses a significant challenge, particularly the continued provision of reliable technical operations and a governance structure capable of responding adequately to user demands. re3data has found suitable solutions to these challenges, which have enabled the service to be in operation for more than ten years. The long-standing collaboration with DataCite has contributed to this success. Participation in third-party-funded projects has facilitated the collaborative development of core service elements together with partners. Participation in committees such as those surrounding EOSC and RDA, as well as active engagement with the RDR community, have motivated discussions about changing requirements and led to the continuous evolution of the registry.

Responsibilities for specific tasks are divided among several entities, such as a working group responsible for guiding future directions of the service and the editorial board responsible for maintaining re3data metadata. In addition, there are teams responsible for technology as well as for outreach and communication. The working group includes experts from DataCite and other stakeholders, who discuss current requirements, prioritize developments, and ensure coordination with RDR operators worldwide. In addition to these entities, coordination with third-party-funded projects involving re3data is ongoing.

#### Lessons learned

Continuous and agile development addresses the users’ constantly evolving needs. Operating a registry that meets those needs in the long term requires flexibility.

### Sustainability

#### Scope

For re3data, sustainability means ensuring a long-term and reliable service to the global RDR community.

#### Approach

Maintaining the sustainable operation of a service like re3data beyond an initial project phase is a challenge. For re3data, the consortium model has proven effective, as the service is supported by a wide range of scientific institutions. This model, which is embedded in the governance of re3data, allows the operation of the service to be sustained through self-funding while also enabling important developments to be undertaken within the scope of third-party projects. Thanks to funding received from the DFG (re3data COREF project) and the European Union’s Horizon 2020 program (FAIRsFAIR project), significant investments have been made in the IT infrastructure and overall advancement of the service in recent years.

#### Lessons learned

A strategy based on diverse revenue streams contributes to securing funding for the service long-term.

### Policies

#### Scope

For re3data, being mentioned in policies comes with a responsibility for operating a reliable service and maintaining high-quality metadata for the global RDR community.

#### Approach

During the development of the re3data service, the partners engaged in dialogues with various stakeholders that were interested in using the registry to refer to RDRs in their policies. They might do this, for example, to recommend or mandate the use of RDRs in general for publishing research data, or the use of a specific RDR. Today, re3data is mentioned in the policies of several funding agencies, scientific institutions, and journals. These actors use re3data to identify RDRs operated by specific academic institutions that were developed using funding from a funding organization, or that store data that are the basis of a journal article. Examples of policies and policy guidance documents that refer to re3data:

- Academic institutions:
  - Brandon University, Canada^59^
  - Technische Universität Berlin, Germany^60^
  - University of Edinburgh, United Kingdom^61^
  - University of Eastern Finland^62^
  - Western Norway University of Applied Sciences^63^
- Funders:
  - Bill & Melinda Gates Foundation, USA^64^
  - European Commission^65^ and ERC, EU^66^
  - National Science Foundation (NSF), USA^67^
  - NIH, USA^68^
- Journals and Publishers:
  - Taylor & Francis, United Kingdom^69^
  - Springer Nature, United Kingdom^70^
  - Sage, United Kingdom^71^
  - Wiley, Germany^72^

Regular searches are conducted to track mentions of re3data in policies. On the re3data website, a list of policies referring to re3data is maintained and regularly updated^73^.

As a result of being mentioned in policies so frequently, re3data receives inquiries from researchers for information on listed RDRs almost daily. These inquiries are usually forwarded to the RDR directly.

#### Lessons learned

Policies represent firm support for research data management by academic institutions, funders, and journals and publishers. By facilitating the search for and referencing of RDRs in policies, re3data further promotes Open Science practices.

### Data reuse

#### Scope

For re3data, data reuse is one of the main objectives, ensuring that third parties can rely on re3data metadata to build services that support the global RDR community.

#### Approach

Because re3data metadata are published as open data, third parties are free to integrate it into their systems. Several service operators have already taken advantage of this opportunity. In general, there are three types of services that work with re3data data:

1. Services for finding and describing RDRs: These services usually work with a subset of re3data metadata. Sometimes, the data is manually curated, and then integrated into external services based on specific parameters. Examples include:
  DARIAH-EU has developed its Data Deposit Recommendation Service based on a subset of re3data metadata, which helps humanities researchers find suitable RDRs^74,75^.
  The American Geophysical Union (AGU) has utilized re3data metadata to create a dedicated gateway for RDRs in the geosciences with its Repository Finder tool^76,77^, which was later incorporated into the DataCite Commons web search interface.
2. Services for monitoring the landscape of RDRs: These services analyze re3data metadata using specific parameters and visualize the results. Examples include:
  OpenAIRE has integrated re3data metadata into its Open Science Observatory to provide information on RDRs that are part of OpenAIRE^78^.
  The European Commission operates the Open Science Monitor, a dashboard that analyzes re3data metadata. The following metrics are displayed: number of RDRs by subject, number of RDRs by access type, and number of RDRs by country^79,80^.
3. Services for assessing RDRs: These services use re3data metadata and other data sources to evaluate RDRs more comprehensively. Examples include:
  The F-UJI Automated FAIR Data Assessment Tool is a web-based service that assesses the degree to which individual datasets conform to the FAIR Data principles. The tool utilizes re3data metadata to evaluate characteristics of the RDR that store the datasets^81^.
  Charité Metrics Dashboard, a dashboard on responsible research practices from the Berlin Institute of Health at Charité in Berlin, Germany, builds on F-UJI data and combines this information with additional re3data metadata^82^.

These examples underscore the value Open Science tools like re3data generate by making their data openly available without restrictions. As a result of the permissive licensing, re3data metadata can be used for new and innovative applications, establishing re3data as a vital data provider for the global Open Science community.

#### Lessons learned

Permissive licensing and extensive collaboration have turned re3data into a key data provider in the Open Science ecosystem.

### Metadata for research

#### Scope

For re3data, providing RDR descriptions also means offering metadata that enables analyses of the global RDR community.

#### Approach

In research disciplines studying data infrastructures, for example library and information science or science and technology studies, re3data is regularly used for information on the state of research infrastructures. As re3data has been mapping the landscape of data infrastructures for ten years, it has evolved into a tool that is used for monitoring Open Science activities, research data management, and other topics. Studies reusing re3data metadata include analyses of the overall RDR landscape, the landscape of RDRs in a specific domain, or the RDR landscape of a region or country. Some examples of studies reusing re3data metadata for research are:

- Overall studies: Boyd^83^ examined the extent to which RDR exhibit properties of infrastructures. Khan & Ahangar^84^ and Hansson & Dahlgren^85^ focused on the openness of RDRs from a global perspective.
- Regional studies: Bauer *et al*.^86^ examined Austrian RDRs, Cho^87^ Asian RDRs, Milzow *et al*.^88^ Swiss RDRs, and Schöpfel^89^ French RDRs.
- Domain studies: Gómez *et al*.^90^ and Li & Liu^91^ investigated the landscape of RDRs in humanities and social science. Prashar & Chander^92^ focused on computer science.

Members of the re3data team have also published studies reusing re3data metadata, including studies of the global state of RDR^93^, openness^39^, and quality assurance of RDRs^41^.

In response to the demand for information on the RDR landscape, the re3data graphical user interface provides various visualizations of the current state of RDRs. For example, re3data metadata can be browsed visually by subject category and on a map. In addition, the metrics page of re3data shows how RDRs are distributed across central properties of the metadata schema^94^.

The start page of re3data includes a recommendation for how to cite the service if it was used as a source in papers:

re3data - Registry of Research Data Repositories. 10.17616/R3D last accessed: [date].

In citing the service, the use of re3data as a data source in research and the service in general becomes more visible.

#### Lessons learned

The increasing number of studies reusing re3data metadata shows a real demand for reliable information on the global RDR landscape.

### Communications

#### Scope

For re3data, communication means engaging in dialogue with relevant stakeholders in the global RDR community.

#### Approach

Broad-based public relations are very important for a service catering to a global community. In recent years, re3data has pursued a communication strategy that includes the following elements:

- Conference presentations: It has been proven effective to represent the service at conferences, paving new ways to engage with the community.
- Mailing lists: The re3data team regularly informs members of a variety of mailing lists about news from the service.
- Social media: re3data communicates current developments via Mastodon (https://openbiblio.social/@re3data) and Twitter (https://twitter.com/re3data).
- Help desk: Communication via the help desk is essential for the re3data service. The help desk team answers questions about RDR descriptions, as well as general questions about data management. The number of general inquiries, e.g., for finding a suitable RDR, has increased over the years.
- Blog: The project re3data COREF operates a blog that informs about developments in the project^95^. Some blog posts are also published in the DataCite Blog^96^.

#### Lessons learned

Establishing broad-based communication channels enables the service to reach and engage with relevant stakeholders in a variety of formats.

## Discussion

Over the past ten years, re3data has evolved into a reliable and valuable Open Science service. The service offers high-quality RDR descriptions from all disciplines and regions. re3data is managed cooperatively; new features are developed in third-party projects.

Four basic principles guide the development of re3data: openness, community engagement, high-quality metadata, and ongoing consideration of users’ needs. These principles ensure that the activities of the service align with the values and interests of its stakeholders. In the context of these principles, ten key issues for the operation of the service have emerged over the last ten years.

In the past two years, following in-depth conversations with diverse parties, a new conceptual model for re3data was developed^45^. This process contributed to a better understanding of the needs of RDR operators and other stakeholders. The conceptual model will guide developments of re3data, embedding the service further in the evolving ecosystem of Open Science services with the intention to support researchers, scientific institutions, funding organizations, publishers, and journals in implementing the FAIR principles and realizing an interconnected global research data ecosystem.

## Methods

This article describes the history and current status of the global registry re3data. Based on operational experience, it reflects on some of the basic principles that have shaped the service since its inception.

Having been launched more than ten years ago, re3data is now the most comprehensive registry of RDRs. The service currently describes more than 3,000 RDRs and caters to a diverse user base including RDR operators, researchers, funding agencies, and publishers. Ten key issues that are relevant for operating an Open Science service like re3data are identified, discussed, and reflected: openness, quality assurance, community engagement, interoperability, development, sustainability, policies, data reuse, metadata for research, and communications. For each of the key issues, we provide a definition, explain the approach applied by the re3data service, and describe what the re3data team learned from working on each issue.

Among other aspects, the paper outlines the design, governance, and objectives of re3data, providing important background information on a service that has evolved into a central data source on the global RDR landscape.

## Data Availability

The re3data RDR descriptions are openly available via https://re3data.org under a CC0 deed.

## Code availability

The source code of the directory is not publicly released. The re3data subject ontology and several Jupyter notebooks with examples for using the re3data API can be found at: https://github.com/re3data.
